# Efficacy of left subclavian artery revascularization strategies during thoracic endovascular aortic repair in patients with type B dissection: A single-center experience of 105 patients

**DOI:** 10.3389/fcvm.2023.1084851

**Published:** 2023-04-03

**Authors:** Xiangyang Wu, Yongnan Li, Yinglu Zhao, Yilin Zhu, Shixiong Wang, Qi Ma, Debin Liu, Bingren Gao, Shilin Wei, Weifan Wang

**Affiliations:** ^1^ Department of Cardiac Surgery, Lanzhou University Second Hospital, Lanzhou University, Lanzhou, China; ^2^ Department of Cardiac Surgery, Hainan General Hospital, Hainan, China; ^3^ Department of Thoracic Surgery, Lanzhou University Second Hospital, Lanzhou University, Lanzhou, China

**Keywords:** type B aortic dissection, endovascular repair, left subclavian artery, revascularization, LSA reconstruction

## Abstract

**Background:**

Left subclavian artery (LSA) revascularization during thoracic endovascular aortic repair (TEVAR) is necessary to reduce postoperative complications in patients with Stanford type B aortic dissection and an insufficient proximal anchoring area. However, the efficacy and safety of different LSA revascularization strategies remain unclear. Here, we compared these strategies to provide a clinical basis for selecting an appropriate LSA revascularization method.

**Methods:**

In this study, we included 105 patients with type B aortic dissection who were treated using TEVAR combined with LSA reconstruction in the Second Hospital of Lanzhou University from March 2013 to 2020. They were divided into four groups according to the method used for LSA reconstruction, namely, carotid subclavian bypass (CSB; *n* = 41), chimney graft (CG; *n* = 29), single-branched stent graft (SBSG; *n* = 21), and physician-made fenestration (PMF; *n* = 14) groups. Finally, we collected and analyzed the baseline, perioperative, operative, postoperative, and follow-up data of the patients.

**Results:**

The treatment success rate was 100% in all the groups, and CSB + TEVAR was the most commonly used procedure in emergency settings compared with the other three procedures (*P* < 0.05). The estimated blood loss, contrast agent volume, fluoroscopic time, operation time, and limb ischemia symptoms during the follow-up were significantly different in the four groups (*P* < 0.05). Pairwise comparison among groups indicated that the estimated blood loss and operation time in the CSB group were the highest (adjusted *P* < 0.0083; *P* < 0.05). The contrast agent volume and fluoroscopy duration were the highest in the SBSG groups, followed by PMF, CG, and CSB groups. The incidence of limb ischemia symptoms was the highest in the PMF group (28.6%) during the follow-up. The incidence of complications (except limb ischemia symptoms) during the perioperative and follow-up periods was similar among the four groups (*P* > 0.05) The median follow-up time of CSB, CG, SBSG, and PMF groups was significantly different (*P* < 0.05), and the CSB group had the longest follow-up.

**Conclusion:**

Our single-center experience suggested that the PMF technique increased the risk of limb ischemia symptoms. The other three strategies effectively and safely restored LSA perfusion in patients with type B aortic dissection and had comparable complications. Overall, different LSA revascularization techniques have their advantages and disadvantages.

## Introduction

Aortic dissection (AD) is a life-threatening disease with an incidence of 35 cases per 100,000 people per year in patients aged 65–75 years (1). The Stanford type B dissection accounts for 25%–40% of all aortic dissections (2, 3). Since the description of thoracic endovascular aortic repair (TEVAR) by Dake et al. (4), several authors have reported the treatment of type B aortic dissections (TBADs) using TEVAR with favorable mid- and long-term outcomes (5–7). Moreover, the 2014 ESC guidelines recommend TEVAR as a first-line treatment for complicated TBADs (8).

Although TEVAR has revolutionized the treatment of TBADs, a minimum of 15 mm of the normal aortic wall is necessary to adequately fix stent grafts (9). However, the proximal seal zone is of inadequate length in 26%–40% of patients (10). In such cases, intentional coverage of the left subclavian artery (LSA) is often performed to extend the sealing zone. Nevertheless, the risk of serious complications, such as stroke, upper extremity ischemia, and spinal cord ischemia (SCI), increases with the coverage of LSA (11–13). Although LSA coverage is tolerated by some patients (14), the latest clinical trials and meta-analyses have revealed that a conventional LSA reconstruction could significantly decrease the risk of postoperative stroke and SCI (15–17).

The Society for Vascular Surgery practice guidelines recommend conventional LSA reconstruction in selected patients (18). However, the recommendations did not suggest the most effective technique for LSA revascularization. Chimney graft (CG), single-branched stent graft (SBSG), physician-made fenestration (PMF), and carotid–subclavian bypass (CSB) are the main methods for LSA revascularization. Here, we summarized our experience of TEVAR with CG, SBSG, PMF, and CSB performed on patients with type B aortic dissections involving the LSA and evaluated their perioperative and follow-up parameters.

## Methods

This single-center retrospective cohort study was initiated by the Department of Cardiac and Vascular Surgery, Second Affiliated Hospital of Lanzhou University. A total of 350 TEVAR surgeries were performed from March 2013 to 2020, and 150 of them were performed on patients with type B aortic dissection and involved LSA management. The management strategy was determined by aortic anatomy, surgeon, and patients following strict inclusion and exclusion criteria. Patients were included based on the following criteria: (I) diagnosed with TBAD using computed tomography angiography (CTA); (II) diagnosed with an insufficient proximal landing zone (the entry tear located <15 mm distal to the LSA) using CTA; (III) no advanced kidney or liver disease; and (IV) no serious anatomic variation. Patients were excluded based on the following criteria: (I) Stanford type A aortic dissection; (II) a penetrating aortic ulcer; (III) a serious artery anatomic variation; (IV) a previous history of TEVAR; (V) severe kidney or liver disease; (VI) allergy to iodine contrast media; (VII) connective tissue disease. such as Marfan syndrome; and (VIII) a postoperative follow-up of <12 months.

**Figure 1:**
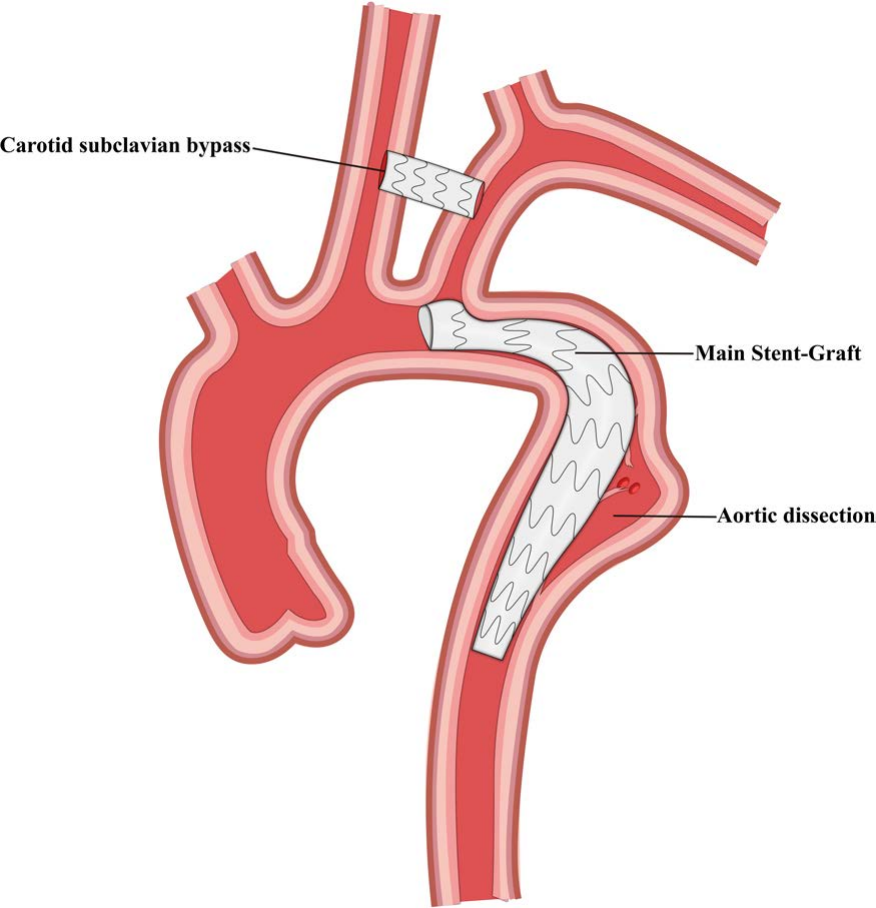
Schematic representation of carotid subclavian bypass and thoracic endovascular aortic repair.

**Figure 2:**
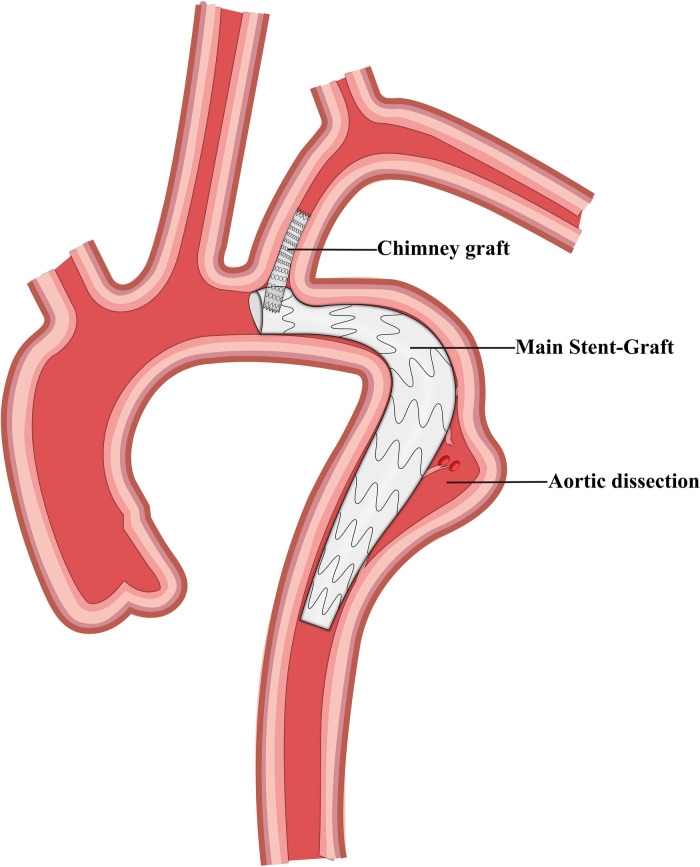
Schematic representation of the chimney graft and thoracic endovascular aortic repair.

**Figure 3:**
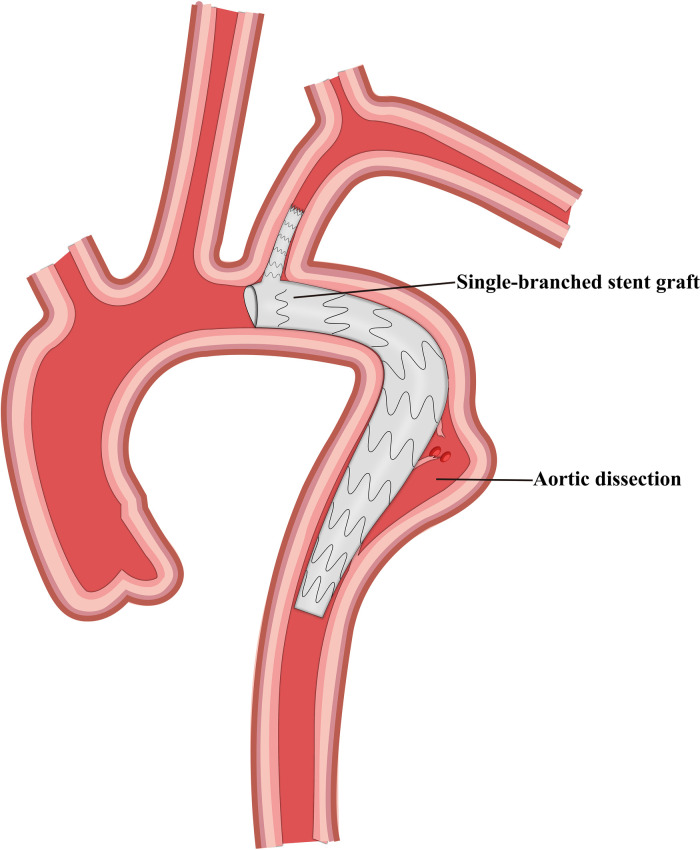
Schematic representation of the single-branched stent graft and thoracic endovascular aortic repair.

**Figure 4:**
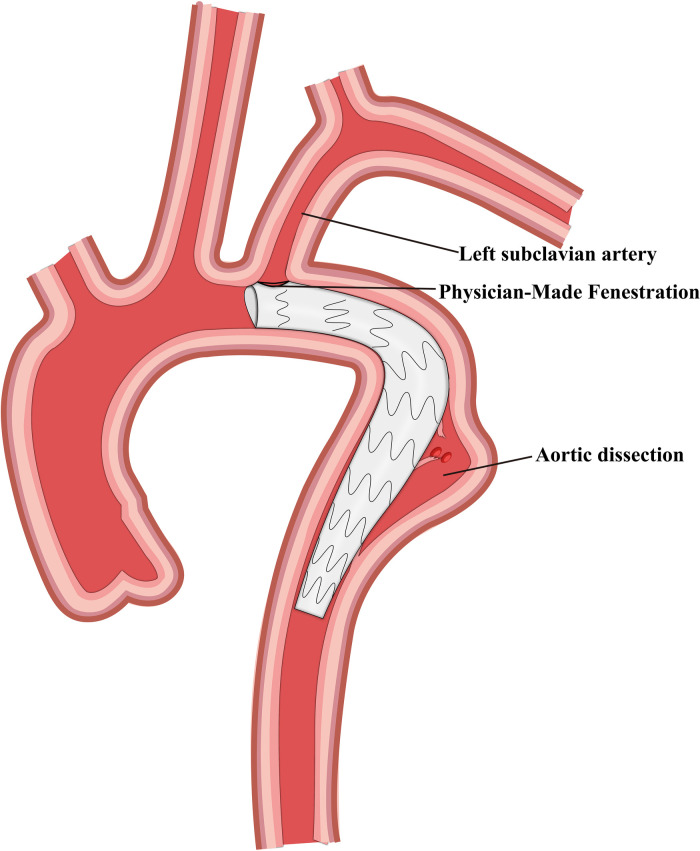
Schematic representation of physician-made fenestration and thoracic endovascular aortic repair.

Finally, we selected 105 patients with TBAD who were treated using TEVAR and LSA revascularization. These patients were divided into four groups based on the revascularization strategy used, and 41, 29, 21, and 14 patients were included in the CSB, CG, SBSG, and PMF groups, respectively (Figures 1–4). Informed signed consent was obtained from each patient included in this study, and the study protocol was approved by the Institutional Ethics Committee of the Second Affiliated Hospital of Lanzhou University (ID: 2021A-016).

Patient information, including demographics, procedural data, and outcomes, was obtained from the electronic medical records for further data analysis. The outcomes of this study included in-hospital mortality, stroke, all-cause mortality, LSA steal syndrome, and procedure-related reintervention. All preoperative variables, including cohort characteristics, and procedural variables, such as procedure type, characteristics, and timing, were studied to assess the difference between groups. All outcomes were defined using standard guidelines (8, 19).

### Statistical analysis

SPSS 26.0 software was used for statistical analysis. Kolmogorov–Smirnov tests were performed to check data normality. Continuous variables were expressed as mean ± standard deviation. Comparisons between multiple groups were performed using a one-way analysis of variance (one-way ANOVA) when the variances were homogeneous. Comparisons between the two groups were performed with Tukey's test. When the variances were nonhomogeneous, we used the Games–Howell test. Continuous variables were expressed as medians and interquartile ranges if data were not normally distributed. Categorical variables were expressed as numbers and percentage frequencies. Comparisons between multiple groups were performed using the Kruskal–Wallis *H* test for categorical variables. The Bonferroni correction was applied to comparisons between the two groups. Comparisons between groups were performed using the chi-square test or Fisher's exact test. *P* < 0.05 was considered statistically significant, and *P* < 0.0083 was considered statistically significant after the Bonferroni correction.

## Results

### Baseline and preoperative characteristics of patients

A total of 105 patients, who met our inclusion criteria, were included in this study, including 41 patients in the CSB group, 29 patients in the CG group, 21 patients in the SBSG group, and 14 patients in the PMF group. The demographic information of patients is presented in Table 1. We did not observe any significant difference in the age (*P* = 0.187), sex (*P* = 0.675), and weight (*P* = 0.338) of the patients included in the four groups. Similarly, the incidence of preoperative comorbidities, including hypertension (*P* = 0.694), hyperlipidemia (*P* = 0.835), diabetes mellitus (*P* = 0.972), and coronary artery disease (*P* = 0.987), was not significantly different among the groups. The preoperative characteristics, including symptoms, NYHA class, aortic regurgitation, mitral regurgitation, pulmonary hypertension, aortic diameters, LSA diameters, and LSA–LCCA distance, were not significantly different among the four groups (Table 2). However, emergency surgery was more frequent in the CSB group (Table 3), and we found that the rate of emergency surgery was significantly higher in the CSB group than that in the SBSG group (adjusted *P* = 0.007).

**Table 1:** Baseline demographics and comorbidities of patients in different treatment groups.

Variables	CSB (*n* = 41)	CG (*n* = 29)	SBSG (*n* = 21)	PMF (*n* = 14)	*P*-value
Male sex	31 (75.6%)	21 (72.4%)	18 (85.7%)	12 (85.7%)	0.675^a^
Age (years)	54.0 (47.5–63.5)	57.0 (51.5–66.5)	51.0 (47–55.5)	49.0 (44.8–66.0)	0.187^b^
Weight (kg)	68.85 ± 9.39	67.85 ± 11.44	72.79 ± 9.48	70.79 ± 9.89	0.338^c^
**Comorbidities**					
Hypertension	33 (80.5%)	26 (89.7%)	17 (81.0%)	11 (78.6%)	0.694^a^
Hyperlipidemia	15 (36.6%)	8 (27.6%)	6 (28.6%)	5 (35.7%)	0.835^d^
Diabetes mellitus	10 (24.4%)	6 (20.7%)	4 (19.0%)	3 (21.4%)	0.972^a^
CAD	6 (14.6%)	5 (17.2%)	3 (14.3%)	2 (14.3%)	0.987^a^
COPD	3 (7.3%)	1 (3.4%)	1 (4.8%)	0 (0%)	0.855^a^
CKD	1 (2.4%)	1 (3.4%)	0 (0%)	0 (0%)	0.648^a^
CVA	2 (4.9%)	2 (6.9%)	1 (4.8%)	1 (7.1%)	0.967^a^
Arrhythmia	11 (26.8%)	4 (13.8%)	4 (19.0%)	3 (21.4%)	0.633^a^
Myocardial infarction	1 (2.4%)	1 (3.4%)	0 (0%)	1 (7.1%)	0.645^a^
PAD	4 (9.8%)	1 (3.4%)	0 (0%)	0 (0%)	0.379^a^
Previous heart surgery	1 (2.4%)	2 (6.9%)	1 (4.8%)	0 (0%)	0.896^a^
Smoking	21 (51.2%)	18 (62.1%)	12 (57.1%)	8 (57.1%)	0.654^d^
Drinking	12 (29.3%)	10 (34.5%)	9 (42.9%)	7 (50.0%)	0.498^d^

Continuous data are shown as means (standard deviations) or medians (interquartile ranges), and categorical data are shown as numbers (%). CAD, coronary heart disease; COPD, chronic obstructive pulmonary disease chronic; CKD, chronic kidney disease; CVA, cerebrovascular accident; PAD, peripheral vascular disease. *P* < 0.05 was considered statistically significant.

^a^ Fisher's exact test.

^b^ Kruskal–Wallis *H* test.

^c^ Tukey's test.

^d^ Pearson’s chi-square test.

**Table 2:** Preoperative characteristics of patients in different treatment groups.

Variable	CSB (*n* = 41)	CG (*n* = 29)	SBSG (*n* = 21)	PMF (*n* = 14)	*P*-value
Chest or back pain	32 (78.0%)	22 (75.9%)	17 (81.0%)	11 (78.6%)	0.986^a^
**NYHA class**					
I	28 (68.3%)	18 (62.1%)	18 (85.7%)	9 (64.3%)	0.320^b^
≥II	13 (31.7%)	11 (37.9%)	3 (14.3%)	5 (35.7%)	
**Preoperative echocardiography**					
Aortic regurgitation	13 (31.7%)	8 (27.6%)	4 (19.0%)	4 (28.6%)	0.784^a^
Mitral regurgitation	12 (29.3%)	6 (20.7%)	4 (19.0%)	5 (35.7%)	0.618^b^
Pulmonary hypertension	4 (9.8%)	2 (6.9%)	2 (9.5%)	2 (15.4%)	0.847^a^
LVEF (%)	60.0 (58.4–65.0)	61.0 (60.0–63.0)	63.0 (58.5–66.0)	60.5 (58.5–64.5)	0.892^c^
**Preoperative CTA**					
Aortic diameter (mm)	29.0 (27.0–31.0)	28.0 (26.0–30.0)	29.0 (27.0–30.0)	28.5 (26.8–30.3)	0.255^c^
LSA diameter (mm)	9.5 (9.0–10.0)	9.5 (9.0–10.5)	10.0 (9.0–11.0)	9.0 (8.5–10.0)	0.176^c^
LSA–LCCA distance (mm)	10.0 (9.5–11.0)	10.5 (10.0–11.5)	11.0 (10.0–12.0)	10.5 (10–11.3)	0.149^c^
**Urgency**					
Emergency	15 (36.6%)	8 (27.6%)	1 (4.8%)	3 (21.4%)	0.042^a^
Elective	26 (63.4%)	21 (72.4%)	20 (95.2%)	11 (78.6%)	

Continuous data are shown as medians (interquartile ranges), and categorical data are shown as numbers (%). CTA, computed tomography angiography; LVEF, left ventricle ejection fraction; LSA, left subclavian artery; LCCA, left common carotid artery; *P* < 0.05 was considered statistically significant.

^a^ Fisher's Exact test.

^b^ Pearson Chi-Square test.

^c^ Kruskal–Wallis *H* test.

**Table 3:** Comparison between two treatment groups.

Variable		CSB vs. CG	CSB vs. SBSG	CSB vs. PMF	CG vs. SBSG	CG vs. PMF	SBSG vs. PMF
Emergency	Adjusted *P*	0.430	0.007	0.297	0.061	1.000	0.129
EBL	Adjusted *P*	<0.001	<0.001	<0.001	0.007	0.033	0.277
Symptoms of limb ischemia	Adjusted *P*	1.000	0.545	0.031	0.503	0.077	0.008
Follow-up time	Adjusted *P*	0.121	<0.001	0.003	0.008	0.248	0.001
Fluoroscopic time	*P*	<0.001	<0.001	<0.001	<0.001	<0.001	<0.001
Amount of contrast	*P*	<0.001	<0.001	<0.001	<0.001	<0.001	<0.001
Operation time	*P*	<0.001	<0.001	0.004	0.241	0.082	0.417

EBL, estimated blood loss. Adjusted *P* < 0.0083 was considered statistically significant; *P* < 0.05 was considered statistically significant.

### Operative details of patients

TEVAR combined with LSA reconstruction was considered successful when the main body of the covered thoracic aortic stent was released successfully, the covered stent isolated the proximal and distal tears of the dissection without complications (including stent distortion or folding and endoleakage), and the patency of the blood flow of the LSA was confirmed. The operative details are summarized in Table 4. TEVAR combined with LSA reconstruction was performed in all groups with a 100% surgical success rate. All procedures were performed under general anesthesia (only one surgery in the CG group was performed under local anesthesia). Stent graft-related complications were not reported during surgery in any of the four groups. The stent graft-related complications, anesthesia, and incidence of endoleakage during surgery did not differ significantly in the four groups; however, the differences in the fluoroscopic time (*P* = 0.001), amount of contrast (*P* < 0.001), estimated blood loss (*P* < 0.001), and operation time (*P* < 0.001) were statistically significant. Comparisons between the two groups are presented in Table 3.

**Table 4:** Operative details of patients in different treatment groups.

Variable	CSB (*n* = 41)	CG (*n* = 29)	SBSG (*n* = 21)	PMF (*n* = 14)	*P*-value
General anesthesia [*n*, (%)]	41 (100%)	28 (96.6%)	21 (100%)	14 (100%)	0.610^a^
Fluoroscopic time (min)	46.62 ± 2.72	55.20 ± 2.42	70.93 ± 3.17	59.55 ± 3.39	0.001^b^
Amount of contrast (ml)	149.76 ± 7.33	200.52 ± 6.49	250.59 ± 7.75	221.51 ± 7.23	<0.001^b^
EBL (ml)	220.0 (210.0–232.5)	90.0 (85.0–102.5)	130.0 (120.0–135.0)	120 (100.0–132.5)	<0.001^c^
Operation time (min)	341.7 ± 78.7	179.7 ± 53.9	221.3 ± 68.1	250.7 ± 88.7	<0.001^d^
Endoleak [*n*, (%)]	0 (0%)	2 (6.9%)	0 (0%)	1 (7.1%)	0.119^a^
SGRC [*n*, (%)]	0 (0%)	0 (0%)	0 (0%)	0 (0%)	–
Surgical success	41 (100%)	29 (100%)	21 (100%)	14 (100%)	–

Continuous data are shown as means (standard deviations) or medians (interquartile ranges), and categorical data are shown as numbers (%). EBL, estimated blood loss; SGRC, stent-graft related complication including fold, twist, and narrow; Tukey's test and Games–Howell test in footnotes b and d only represent the homogeneity of variance test among the four groups, and *P*-value only represents the results of one-way analysis of variance; *P* < 0.05 was considered statistically significant.

^a^ Fisher's exact test.

^b^ Tukey's test.

^c^ Kruskal–Wallis *H* test.

^d^ Games–Howell test.

### In-hospital outcomes after surgery

Postoperative outcomes before discharge are shown in Table 5. In-hospital death, symptoms of limb ischemia, paraplegia, and lymphatic leakage were not reported in any group. The complications, including stroke (transient ischemic attack), hematoma, pneumonia, LSA steal syndrome, neurologic injury, and blood transfusion, did not differ significantly in the four groups. However, one case of stroke occurred in CG and PMF groups. The CSB and CG groups had a case of LSA syndrome that recovered without reintervention. One patient of the CSB group developed symptoms of nerve injury, which may be caused by the injury of the brachial plexus during the surgery. The symptoms were alleviated after conservative treatment during the hospital stay. The duration of stay in the ICU and hospital was not significantly different among the four groups.

**Table 5:** In-hospital outcomes in patients after surgery.

Variable	CSB (*n* = 41)	CG (*n* = 29)	SBSG (*n* = 21)	PMF (*n* = 14)	*P*-value
Mortality	0 (0%)	0 (0%)	0 (0%)	0 (0%)	–
Symptoms of limb ischemia	0 (0%)	0 (0%)	0 (0%)	0 (0%)	–
Stroke	0 (0%)	1 (3.4%)	0 (0%)	1 (7.1%)	0.258^a^
Hematoma	5 (12.2%)	1 (3.4%)	0 (0%)	1 (7.1%)	0.317^a^
Paraplegia	0 (0%)	0 (0%)	0 (0%)	0 (0%)	–
Pneumonia [*n*, (%)]	5 (12.2%)	2 (6.9%)	2 (9.5%)	1 (7.1%)	0.958^a^
LSA steal syndrome [*n*, (%)]	1 (2.4%)	1 (3.4%)	0 (0%)	0 (0%)	1.000^a^
Lymphatic leakage [*n*, (%)]	0 (0%)	0 (0%)	0 (0%)	0 (0%)	–
Neurologic injury [*n*, (%)]	1 (2.4%)	0 (0%)	0 (0%)	0 (0%)	1.000^a^
Blood transfusion [*n*, (%)]	6 (14.6%)	3 (10.3%)	1 (4.8%)	1 (7.1%)	0.647^a^
Stent-associated infection	0 (0%)	0 (0%)	0 (0%)	0 (0%)	–
Reintervention [*n*, (%)]	0 (0%)	0 (0%)	0 (0%)	0 (0%)	–
Length of ICU (h)	36.38 ± 19.17	31.10 ± 20.05	26.55 ± 13.76	29.79 ± 19.31	0.198^b^
Length of hospital stay (days)	9.63 ± 3.46	8.72 ± 3.76	7.71 ± 3.20	9.21 ± 2.49	0.204^b^

Continuous data are shown as means (standard deviations), and categorical data are shown as numbers (%). LSA, left subclavian artery; ICU, intensive care unit; Footnote b only represents the homogeneity of variance test among the four groups, and *P*-value only represents the results of one-way analysis of variance; *P* < 0.05 was considered statistically significant.

^a^ Fisher's exact test.

^b^ Tukey's test.

### Postoperative follow-up outcomes

Overall postoperative follow-up outcomes are summarized in Table 6. Three patients died during postoperative follow-up, and one patient died in each of the CSB, CG, and PMF groups. In the CG group, a patient died 2 months after surgery because of retrograde type A aortic dissection (RAAD). One patient of the PMF group had LSA steal syndrome, and one patient of the CSB group underwent reintervention because of an endoleak. The CTA review showed type IB endoleak, which was resolved by implanting a thoracic aorta-covered stent combined with CUFF-covered stent reintervention. The endoleak disappeared after the intervention, and a complete false lumen thrombosis was observed in the CTA review 1 year later.

**Table 6:** Follow-up outcomes in patients.

Variable	CSB (*n* = 41)	CG (*n* = 29)	SBSG (*n* = 21)	PMF (*n* = 14)	*P*-value
Mortality	1 (2.4%)	1 (3.4%)	0 (0%)	1 (7.1%)	0.648^a^
Symptoms of limb ischemia	2 (4.9%)	2 (6.9%)	0 (0%)	4 (28.6%)	0.023^a^
LSA steal syndrome	0 (0%)	0 (0%)	0 (0%)	1 (7.1%)	0.133^a^
Stroke	2 (4.9%)	2 (6.9%)	1 (4.8%)	1 (7.1%)	0.975^a^
SCI	0 (0%)	0 (0%)	0 (0%)	0 (0%)	–
Endoleak	2 (4.9%)	5 (17.2%)	1 (4.8%)	1 (7.1%)	0.280^a^
RTAD	0 (0%)	1 (3.4%)	0 (0%)	0 (0%)	0.610^a^
Reintervention	1 (2.4%)	0 (0%)	0 (0%)	0 (0%)	1.000^a^
CFLT	37 (90.2%)	25 (86.2%)	18 (85.7%)	11 (78.6%)	0.686^a^
Follow-up time (months)	28.0 (17.0–33.0)	24.0 (16.0–28.0)	15.0 (13.5–17.5)	21.5 (19.0–31.5)	<0.001^b^

Continuous data are shown as medians (interquartile ranges), and categorical data are shown as numbers (%). LSA, left subclavian artery; SCI, spinal cord ischemia; CFLT, complete false-lumen thrombosis; *P* < 0.05 was considered statistically significant.

^a^ Fisher's exact test.

^b^ Kruskal–Wallis *H* test.

The median follow-up durations for CSB and CG groups were 28.0 (IQR: 17.0–33.0) and 24.0 (IQR: 16.0–28.0) months, respectively. The similar durations for SBSG and PMF groups were 15.0 (IQR: 13.5–17.5) and 21.5 (IQR: 19.0–31.5) months, respectively. We found that the follow-up time was significantly shorter in the SBSG group (adjusted *P* < 0.0083) and significantly longer in the CSB group than that in the PMF group (adjusted *P* < 0.0083) (Table 3). The incidence of stroke was 4.9%, 6.9%, 4.8%, and 7.1% in the CSB, CG, SBSG, and PMF groups, respectively. The incidence of stroke and the rate of complete false lumen thrombosis were not significantly different among the four groups. In addition, spinal cord ischemia was not observed during follow-up in all the groups. However, limb ischemia symptoms were reported, and their prevalence was significantly lower in the SBSG group than that in the PMF group.

## Discussion

The registry data show that TEVAR for thoracic aortic pathologies has more acceptable outcomes and lower perioperative complications than open repair in the last two decades (20, 21). However, TEVAR with intentional LSA coverage for good fixation of stent grafts carries a high risk for left upper extremity ischemia and stroke (22, 23). The European Society for Vascular Surgery recommends routine revascularization for elective cases to prevent the devastating neurological consequences of spinal cord ischemia and stroke following TEVAR (18). Nevertheless, the efficiency of different LSA reconstruction strategies remains unclear.

Several new LSA reconstruction strategies are constantly emerging with the evolution of medical devices and surgical technology. Carotid subclavian bypass, chimney graft, single-branched stent graft, and physician-made fenestration are the most common surgical techniques for reconstructing LSA, and all of these strategies have been used in our center for the treatment of Stanford type B dissection. Here, we summarized our single-center experience of 105 patients who underwent TEVAR combined with four different strategies of LSA reconstruction and compared the efficiency of the four strategies.

We observed a significant difference in the methods used for emergent surgeries. The CSB technique was most commonly used in emergency settings. Bypass technology is the earliest application in LSA reconstruction, and its safety and reliability have been reported in many studies (24, 25). It is especially suitable for emergent cases because of its relatively simple maneuver and wider clinical situations. However, SBSG was not used in emergencies because it was custom-made and involved a long manufacturing time and increased cost. Since the introduction of the CG technique by Criado (26), it has been used to expand proximal landing zones in TEVAR with favorable results (27, 28). However, a gutter between the main graft, aortic wall, and CG can be a potential site for endoleaks after TEVAR (29, 30), and an endoleak is a common complication during surgery. The single-branched stent graft designed by Inoue and colleagues (31) is intended to treat pathologies involving the LSA in the distal arch. Several clinical trials on SBSG have demonstrated favorable short-term results in aortic arch reconstruction (32, 33). Compared to the chimney technique, SBSG implantation is less likely to cause endoleaks because there is no risk of gutter formation. We observed two cases of endoleaks during surgery in the CG group (incidence rate: 6.9%). Although a small endoleak happened in a patient of the PMF group, reintervention was not necessary. Similar to our study, Zhang et al. compared CG and SBSG in the same situation and did not report any significant differences (34). Moreover, the difference in endoleak occurrence was not statistically different among the four groups.

We found a significant difference in the fluoroscopic time and amount of contrast among the four groups. The values were highest in the SBSG group, followed by PMF, CG, and CSB groups. CSB was associated with the lowest fluoroscopic time and amount of contrast because all other three techniques required more time and contrast to confirm the patency of LSA. Moreover, the position of the LSA stent needed to be adjusted according to the results of intraoperative angiography. Compared with the other three groups, the CSB group was associated with more estimated blood loss during surgery. This happens because CSB combines open surgery and endovascular repair. However, our data suggested that the estimated blood loss was much less than that reported by D'Oria (VQI data: 220 vs. 309 ml) (35). The CSB group had a significantly longer operation time than the other three groups, which could be attributed to the lack of a hybrid operation room in our center and the necessity of transferring patients.

The in-hospital outcomes were not significantly different among the groups; however, there was a significant difference in the incidence of limb ischemia symptoms. We found that the PMF group was associated with a much higher rate of symptoms of limb ischemia compared with the SBSG group (28.6% vs. 0%; *P* = 0.008). The PMF technique for aorta repair offers a more judicious approach with favorable mid-term results without altering the anatomic structures (36, 37). Nevertheless, the target branch needs to be at a vertical angle to the aortic arch for using this technique, and tortuosity and the sharp angle of the branch vessels could significantly raise the procedural challenges. Therefore, its long-term efficiency should be confirmed in further studies. In this study, the follow-up time was significantly longer in the PMF group than that in the other groups, and we assumed that a relatively short follow-up might be associated with a lower incidence of symptoms of limb ischemia.

RAAD is a serious TEVAR-related complication. Its incidence ranges from 2.5% to 10%, and the perioperative mortality is >40% (38–40). We had one case of RAAD in the CG group (incidence rate: 3.4%), which resulted in the death of the patient during follow-up. Several predictive risk variables are linked to RAAD, including the timing of TEVAR, the size of the ascending aorta, the proximal landing zone, the extent of false lumen thrombosis, and the proximal-neck balloon dilatation (41–43). The MOTHER registry analysis revealed that RAAD occurred more frequently when the patients were treated in the acute phases and when the stent graft was noticeably oversized (44). In our experience, the preoperative CT-based graft oversizing was limited to 110% to prevent further stress on the aortic wall.

### Limitations

This study has some possible limitations. We designed this study as a single-center retrospective study. The LSA reconstruction strategy depends on the judgment of the surgeon and the subjective initiative of the patient, which could cause selection bias. The results need to be further verified by multicenter randomized controlled studies. In addition, the number of enrolled patients was small with a relatively short follow-up; therefore, a larger number of patients with long-term follow-up should be included in further studies.

## Conclusion

Our single-center experience suggested that the PMF technique was associated with a higher risk of symptoms of limb ischemia, and the other three strategies effectively and safely restored LSA perfusion with comparable complications in patients with TBAD. LSA revascularization techniques have their unique advantages and disadvantages. Although CSB increases operation time and intraoperative blood loss, it is more suitable for emergencies. However, our results should be further verified in a multicenter randomized controlled study with a larger sample size.

## Data Availability

The raw data supporting the conclusions of this article will be made available by the authors without undue reservation.
